# Sodium-to-Potassium Ratio as an Indicator of Diet Quality in Healthy Pregnant Women

**DOI:** 10.3390/nu14235052

**Published:** 2022-11-27

**Authors:** Martina Vulin, Lucija Magušić, Ana-Maria Metzger, Andrijana Muller, Ines Drenjančević, Ivana Jukić, Siniša Šijanović, Matea Lukić, Lorena Stanojević, Erna Davidović Cvetko, Ana Stupin

**Affiliations:** 1 Department of Obstetrics and Gynecology, University Hospital Osijek, HR-31000 Osijek, Croatia; 2 Department of Obstetrics and Gynecology, Faculty of Medicine, Josip Juraj Strossmayer University of Osijek, HR-31000 Osijek, Croatia; 3 Department of Physiology and Immunology, Faculty of Medicine, Josip Juraj Strossmayer University of Osijek, HR-31000 Osijek, Croatia; 4 Scientific Center of Excellence for Personalized Health Care, Josip Juraj Strossmayer University of Osijek, HR-31000 Osijek, Croatia; 5 Faculty of Medicine, Josip Juraj Strossmayer University of Osijek, HR-31000 Osijek, Croatia; 6 Lavoslav Ružička College of Applied Sciences of Vukovar, HR-32000 Vukovar, Croatia

**Keywords:** pregnancy, sodium-to-potassium ratio, micronutrients, gestational weight gain, 24 h urine, food frequency questionnaire

## Abstract

This study aimed to investigate diet quality in healthy pregnant women based on the Na-to-K ratio from 24 h urine sample and food frequency questionnaire (FFQ), to compare dietary micro- and macronutrient intake with current nutritional recommendations (RDA), and to investigate whether gestational weight gain (GWG) is associated with Na-to-K ratio and diet quality during pregnancy in general. Sixty-four healthy pregnant women between 37 and 40 weeks of gestation participated in the study. Participants’ GWG, body composition, molar 24 h urine Na-to-K ratio, and FFQ data on average daily total energy, food groups, and micro-/macronutrient intake were obtained. A Na-to-K ratio of 2.68 (1.11–5.24) does not meet nutrition quality and is higher than the WHO recommendations due to excessive sodium and insufficient potassium intake. FFQ Na-to-K ratio was associated with a higher daily intake of soups, sauces, cereals, fats, and oils and a low intake of fruit and non-alcoholic beverages. A total of 49% of pregnant women exhibited excessive GWG, which was attributed to the increase in adipose tissue mass. GWG was not associated with total energy but may be the result of insufficient physical activity during pregnancy. Daily intake of vitamin D, vitamin E, folate, niacin, riboflavin, calcium, iron, and zinc was suboptimal compared to RDA.

## 1. Introduction

Over the past decades, nutrition has been revealed as a critical determinant of population health, especially because of the clear connection between specific environmental changes and the increased prevalence of noncommunicable diseases [1]. According to the ‘fetal programming’ hypothesis, an appropriate diet during pregnancy is considered fundamental for the health of both mother and child [2,3]. Moreover, the results of both clinical and research studies have associated maternal diet with adverse pregnancy outcomes, e.g., deficiencies in macro- and micronutrients can affect maternal health, pregnancy outcome, and neonatal wellbeing [2].

Based on metabolic effects, nutrients are categorized into macro- and micronutrients. Macronutrients are required in larger amounts; they provide calories that provide energy to the body and are essential for growth and repair [4]. Excessive energy consumption (that surpasses the energy requirements) leads to obesity, diabetes, heart diseases, and other metabolic syndromes. Energy intake during pregnancy depends on individual body mass index and physical activity [5]. IOM estimates that during the third trimester, energy requirements increase by 452 kcal/day, with 45–65% of total energy originating from carbohydrates, 10–35% from protein, and 20–35% from fats [6]. Recommended micronutrient intake is determined by the ‘recommended dietary allowances’ (RDA). Micronutrients that are considered as most beneficial during pregnancy are iron, folic acid, iodine, calcium, zinc, n-3 polyunsaturated fatty acids (n-3 PUFAs), vitamin D, C, E, and B-complex [7]. Food sources rich in micronutrients, especially in potassium, are vegetables (leafy greens, beans, starchy vegetables), fruits, nuts, eggs, and dairy products, while the highest sources of dietary sodium are packaged, processed, store-bought, ready-made foods, bread, butter and oil-based spreads [8]. According to data from large epidemiological studies, potassium-rich food is less often consumed than the one rich in sodium in general, which could also lead to poor micronutrient dietary intake and deficiency during pregnancy. That is the reason why micronutrient supplements are routinely recommended during pregnancy [7]. The leading causes of high sodium and low potassium intake are low awareness of sodium intake, as well as the fact that potassium-rich food is associated with higher costs compared to widely available food rich in sodium [9].

High dietary sodium, together with insufficient potassium intake, is associated with blood pressure (BP) increase and arterial hypertension, as well as the risk of cardiovascular disease (CVD) [10]. In recent years, the sodium-to-potassium (Na-to-K) ratio has been introduced as a more reliable index to assess CVD risk and CVD-related mortality than either sodium or potassium intake alone [11,12]. The World Health Organization (WHO) recommends ingestion of less than 2000 mg of sodium per day and more than 3510 mg of potassium per day [13], resulting in a Na-to-K ratio of ≤1.0, which is believed to be optimal for preserving cardiovascular health. However, considering low compliance to dietary guidelines of sodium and potassium intake and recent results indicating that Na-to-K ratios between 1.0 and 2.0 reduce CVD risk, Na-to-K ratio ≤ 2.0 has been suggested as a suboptimal goal [14].

Gestational weight gain (GWG) is a repercussion of achieving favorable conditions for the growing fetus and consists of maternal body composition and the weight of the fetus, placenta, and amniotic fluid [15]. In the past decade, excessive GWG in pregnancy was recognized as a widespread health issue, especially since inadequate GWG was associated with a higher risk of adverse pregnancy outcomes, such as preterm birth, macrosomia, and cesarean delivery [16]. Various factors have been shown to influence GWG, including body mass index (BMI) before pregnancy, dietary habits, age, socioeconomic status, comorbidities, smoking, etc. [17,18]. Consequently, to a growing number of women living with overweight and obesity at conception and increasing pre-pregnancy BMI, the Institute of Medicine (IOM) has issued recommendations on the optimal GWG [6]. Despite developed and well-established recommendations for adequate GWG during pregnancy, almost 75% of pregnant women do not meet those recommendations but exhibit excess or insufficient GWG [19]. A potential interrelation and association between a Na-to-K ratio as an index of cardiovascular risk and GWG as an indicator of adverse pregnancy outcome risk are yet to be determined.

Thus, the present study aimed to investigate diet quality in healthy pregnant women in the third trimester of pregnancy based on the assessment of the Na-to-K ratio from both 24 h urine sample and food frequency questionnaire (FFQ) and to detect the potential cause for Na-to-K ratio in the observed population. A second goal was a comparison of dietary micro- and macronutrient intake from EPIC-Norfolk FFQ and current nutritional recommendations (RDA) in order to detect whether micro- and macronutrient intake in the observed population correspond to RDA. The third goal was to investigate whether GWG is associated with the Na-to-K ratio and diet quality during pregnancy in general.

## 2. Materials and Methods

### 2.1. Study Population

The present study included sixty-four healthy pregnant women between 37 and 40 weeks of gestation. Participants were recruited during their regular visits to an obstetrician/gynecologist at the Department of Obstetrics and Gynecology, University Hospital Osijek, Osijek, Croatia. Smoking, preconception hypertension, hypertension during pregnancy, thrombophilia, use of low-molecular-weight heparin, coronary heart disease, renal disease, preconception diabetes, gestational diabetes, peripheral artery and CVD, and any other preconception disease affecting vascular and endothelial function were exclusion criteria for participation. Informed consent was obtained from all participants assigned to the study. This study included protocols and procedures that adhered to the standards set by the latest revision of the Declaration of Helsinki. The study was approved by the Ethical Committee of the University Hospital Osijek, Osijek, Croatia (R1/6414/2021), as well as the Ethical Committee of the Faculty of Medicine Josip Juraj Strossmayer University of Osijek, Osijek, Croatia (Cl: 602-04/21-08/07; No: 2158-61-07-21-10). This study is a part of a clinical trial investigating the effects of dietary salt intake during pregnancy on maternal vascular endothelial function in healthy pregnant women, registered at ClinicalTrials.gov (ID NCT05048225 Dietary Salt During Pregnancy and Maternal Vascular Function).

### 2.2. Study Design

The study was designed as a cross-sectional study in which data were obtained between 37 and 40 weeks of pregnancy. On the first visit, participants were informed in detail about the interventions this study involved and provided informed consent. Detailed instructions for a 24 h urine collection and for completing the FFQ were given to the participants. The collected 24 h urine sample and the completed FFQ were obtained, and all further described measurements were performed at the next visit to the obstetrician/gynecologist, which was scheduled 2 to 7 days from the recruitment visit, depending on the duration of gestation. Additionally, participants’ weight was measured again at the time they were admitted to the hospital for delivery.

### 2.3. Body Mass Index, Body Composition, Body Fluid Status, and Arterial Blood Pressure Measurement

Participants’ BMI was calculated from measured height (m) and weight (kg) [20]. GWG was calculated according to the data on the participant’s body weight data before or during early pregnancy that was taken from the pregnancy booklet filled in by the participant’s primary gynecologist and weight measured at the time of admission to the hospital for delivery. To assess whether maternal gestational weight gain (GWG) was low, normal, or high, 2009 IOM recommendations for gestational weight gain during pregnancy were used [6]. The IOM recommendations take into consideration weight gains by pre-pregnancy BMI, according to which normal GWG is considered as follows: BMI < 19.8 kg/m^2^ total weight gain between 12.5 and 18 kg; BMI = 19.8 to 26.0 kg/m^2^ total weight gain between 11.5 and 16 kg; BMI >26.0 to 29.0 kg/m^2^ total weight gain between 7.0 and 11.5 kg; and for BMI > 29.0 kg/m^2^ total weight gain of 7.0 kg [6].

A 4-terminal portable impedance analyzer (Maltron Bioscan 920-II, Maltron International Ltd., Rayleigh, Essex, U.K.) was used for the measurement of body composition and body fluid status. Empirically derived formulas (the original manufacturer’s software) were used to calculate resting metabolic rate (RMR kcal), fat-free mass (FFM%), fat mass (Fat%), total body water (TBW%), extracellular water (ECW%), intracellular water (ICW%), plasma fluid (PF), interstitial fluid (IF), and body density (kg/L).

Arterial blood pressure (BP) was expressed as an average of three consecutive measurements on the same arm. BP measurement was performed after 15 min resting in a seated position using an automated oscillometric sphygmomanometer (OMRON M3, OMRON Healthcare Inc., Osaka, Japan).

### 2.4. 24-h Urine Samples Analysis

The 24 h urine samples were analyzed for sodium, potassium, urea, creatinine coefficient, protein, and albumin concentration. A 24 h urinary sodium and potassium molar excretion was used for the assessment of daily sodium and potassium intake in milligrams (mg) using the appropriate formula (1 mmol = 22.99 mg of sodium or 39.10 mg of potassium). The 24 h urine samples were analyzed at the Clinical Institute of Laboratory Diagnostics, University Hospital Osijek, Osijek, Croatia.

### 2.5. Food Frequency Questionnaire (FFQ)

The publicly available EPIC-Norfolk food frequency questionnaire (https://www.epic-norfolk.org.uk/wp-content/uploads/2020/11/CAMB-PQ-6-1205a_front.pdf (accessed on 5 May 2021)) was used to record the average dietary intake during the previous year in each participant. EPIC-Norfolk FFQ was downloaded, translated to Croatian, cross-culturally adapted, and validated. The questionnaire consists of two parts. Part 1 consists of a food list of 130 lines, with each line having a portion size attached to it. Study participants were requested to select an appropriate frequency of consumption for each line from the nine frequency categories. The FFQ food list was slightly modified in a way that brand names were adapted to those available on the Croatian market. Part 2 contains further questions, which in part ask for more detailed information that links back to food lines in part 1. Obtained data by EPIC-Norfolk FFQ were entered into a spreadsheet, according to the provided instructions, and processed using FFQ EPIC Tool for Analysis (FETA) (https://www.epic-norfolk.org.uk/for-researchers/ffq/ (accessed on 5 May 2021)). FETA provides freely available, stand-alone tools that can produce nutrient and food group intake values from data collected using the EPIC-Norfolk FFQ. FETA produces nutrient output that contains an average daily intake of 46 nutrients and 14 basic food groups [21,22].

Goldberg cut-offs were used to evaluate reported energy intake at the individual level by FFQ [23]. First, the individual ratio between reported energy intake (EIrep) and RMR (EIrep/RMR) was calculated. All participants were categorized as having low physical activity levels (PAL = 1.4). Calculated cut-offs for this PAL category and ages between 18 and 69 years were as follows: lower cut-off 0.872 and upper cut-off 2.249 [24]. The individual EIrep/RMR of each participant was compared with the calculated lower and upper cut-offs, according to which participants were categorized as low energy reporters (LERs) and non-LERs.

### 2.6. Statistical Analysis

All data results were reported as the arithmetic mean and standard deviation (SD). The Shapiro–Wilk normality test was used for the normality of data distribution assessment. When variables were normally distributed, differences between values were tested by Student t-tests. When variables were non-normally distributed, the Mann–Whitney test was used. The correlations between normally distributed variables were tested by Pearson’s correlation test and for non-normally distributed variables by Spearman’s correlation test. *p* < 0.05 was considered statistically significant. Statistical analysis was performed using SigmaPlot, version 11.2 (Systat Software, Inc., Chicago, IL, USA).

## 3. Results

### 3.1. Gestational Weight Gain (GWG)

Sixty-four healthy pregnant women in the third trimester of normal pregnancy were included in the present study (average period of gestation 37.9 ± 0.9 weeks). Participants’ initial body mass index was 23.8 ± 4.3 kg/m^2^, with 2 participants classified as underweight (BMI ≤ 18.5), 45 classified as normal (BMI 18.6–25.0),11 as overweight (BMI 25.1–30.0), and 6 of them as obese (BMI ≥ 30.1). Gestational weight gain (GWG) for participants with BMI ≤ 25.0 kg/m^2^ and for those with BMI >25.1 kg/m^2^ is shown in Table 1. According to 2009 IOM recommendations [6], in participants with initial BMI ≤ 25.1 kg/m^2^, total GWG was low in 7 participants (14.9%), normal in 18 participants (38.3%), and high in 22 participants (48.8%). On the other hand, in participants with initial BMI >25.1 kg/m^2^, total GWG was low in 1 participant (5.9%), normal in 3 participants (17.6%), and high in 13 participants (76.5%). Total GWG did not significantly differ between participants with initial BMI ≤ 25.0 kg/m^2^ and those with BMI > 25.1 kg/m^2^. There was no correlation between GWG and participants’ initial BMI (r = 0.040, *p* = 0.754). Participants’ general characteristics, body mass index, and GWG are described in Table 1.

### 3.2. Body Composition, Body Fluid Status, and Arterial Blood Pressure

Participants’ body composition, body fluid status, and arterial blood pressure values are described in Table 2. Participants’ GWG positively correlated with fat mass (r = 0.323, *p* = 0.010) and negatively correlated with fat-free mass (r = −0.323, *p* = 0.010). In addition, GWG negatively correlated with total body water (r = −0.297, *p* = 0.019) and body density (r = −0.296, *p* = 0.020), indicating that the increase in the proportion of adipose tissue during pregnancy is the most important determinant of weight gain in pregnant women.

All participants had normal systolic BP, diastolic BP, and MAP values and were considered normotensive. There was no significant correlation between GWG and BP values. Diastolic BP positively correlated with extracellular water (r = 0.308, *p* = 0.015), ECW/ICW (r = 0.318, *p* = 0.012), and plasma fluid volume (r = 0.264, *p* = 0.038). There was no correlation between systolic BP and measured body composition and body fluid status parameters.

### 3.3. 24-h Urine

Results of the 24 h urine analysis are presented in Table 3. According to 24 h urine analysis, the average molar Na-to-K ratio was 2.74 ± 0.91. The estimated daily salt (NaCl) intake in healthy pregnant women in the third trimester of pregnancy was 8.2 ± 2.7 g per day. There was no correlation between molar urine Na-to-K ratio and initial BMI, GWG, body composition, body fluid status, and arterial BP.

### 3.4. EPIC-Norfolk Food Frequency Questionnaire (FFQ)

Daily micronutrient intake in healthy pregnant women assessed by EPIC-Norfolk food frequency questionnaire (FFQ) is presented in Table 4. Data obtained by FFQ were compared to RDA for pregnant women. When compared to RDA, FFQ results showed a suboptimal daily intake of vitamin D, vitamin E, folate, iron, and zinc. Daily intake of niacin, riboflavin, thiamin, vitamin B6, vitamin A, potassium, and calcium has been shown as optimal, while daily intake of vitamin B12, vitamin C, sodium, phosphorus, and selenium was higher than the RDA values.

According to daily intake in grams obtained by FFQ, the molar Na-to-K ratio was calculated and was 1.51 ± 0.45. The estimated daily salt (NaCl) intake from FFQ results in healthy pregnant women in the third trimester of pregnancy was 7.53 ± 2.54 g per day.

Daily energy and food group intakes in pregnant women are presented in Table 5. Reported energy intake (EIrep) was significantly below the recommended values for the third trimester of pregnancy (EIrep 1941 ± 638 vs. recommended RMR 2130 ± 93, *p* < 0.001). The average EIrep/RMR ratio was 0.93 ± 0.32, indicating that 42.2% of participants were low energy reporters (LERs). Energy derived from protein (17.9%) was within the recommended interval (10–35% of energy), carbohydrates intake (45.8%) was closer to the lower limit of the recommendations (45–65% of energy), while total fats intake (38.4%) was slightly higher than the recommended reference (20–35% of energy) [2,26].

FFQ Na-to-K ratio positively correlated with higher daily soups and sauces intake (r = 0.475, *p* < 0.001), cereals and cereal products intake (r = 0.454, *p* < 0.001), as well as fats and oils intake (r = 0.291, *p* = 0.022), but negatively correlated with daily fruit (r = −0.336, *p* = 0.008) and non-alcoholic beverages intake (r = −0.284, *p* = 0.025). FFQ molar Na-to-K ratio did not correlate with other daily food group intakes, including alcoholic beverages, eggs and egg dishes, fish and fish products, meat and meat products, milk and milk products, nuts and seeds, potatoes, sugars, and vegetables.

There was no significant correlation between GWG and FFQ molar Na-to-K ratio, total energy or macronutrient intake, or protein, carbohydrates, and fat intake.

### 3.5. Molar Sodium-to-Potassium Ratio

The urinary molar Na-to-K ratio calculated from 24 h sodium and potassium excretion was higher than the FFQ molar Na-to-K ratio calculated from daily sodium and intake (molar Na-to-K ratio urinary vs. FFQ 2.74 ± 0.31 vs. 1.51 ± 0.45, *p* < 0.001). There was no correlation between urinary and FFQ molar Na-to-K ratio in healthy pregnant women (r = 0.229, *p* = 0.073).

## 4. Discussion

The main finding of the present study is that a molar 24 h urinary Na-to-K ratio of 2.74 ± 0.91 does not meet nutrition quality and is higher than the WHO recommendations (urinary molar Na-to-K ratio < 1.33) since recommended value refers to sodium intake < 2000 mg/day and potassium intake > 3500 mg/day. Furthermore, the molar Na-to-K ratio obtained by the FFQ (1.51 ± 0.45) was much closer to the WHO recommendations, but according to the literature, there is still superiority of the assessment of sodium and potassium intake by 24 h urine collection compared to FFQ. According to 24 h urine analysis, such a high Na-to-K ratio seems to be the result of both excessive sodium and insufficient potassium dietary intake. Inadequacy was also observed for micronutrient intake, essential to maintain fetal growth and development [4], showing suboptimal intake of vitamin D, vitamin E, folate, iron, and zinc. In addition, the reported total energy intake in healthy pregnant women in the third trimester of pregnancy was below the recommended values, which is possibly attributed to the 42.2% of participants showing as low energy reporters (LERs). Reported energy intake derived from proteins was within the recommendations, energy derived from carbohydrates was closer to the lower limit of the recommendations, while total fat intake exceeded the recommended reference [2,26]. Only 38% of examined pregnant women with initial BMI ≤ 25.1 kg/m^2^ had weight gain during pregnancy that was considered normal, with concerning data on 47% of them having high weight gain during pregnancy. In addition, only 18% of pregnant women with an initial BMI > 25.1 kg/m^2^ had normal GWG, while 77% of them had high GWG. Body composition analysis indicates that an increase in the proportion of adipose tissue during pregnancy is the most important determinant of GWG in pregnant women. Interestingly, weight gain during pregnancy was not associated with total energy intake according to the FFQ, which can be attributed to underreporting of energy intake from an FFQ (42% of participants classified as LERs). Altogether, present findings on both results of Na-to-K ratio as well as micronutrients intake suggest the inadequate dietary quality of healthy pregnant women, with concerning data of 47% of pregnant women with preconception BMI ≤ 25.0 kg/m^2^ and 77% of those with preconception BMI > 25.1 kg/m^2^ exhibiting excessive GWG during pregnancy, mainly attributed to an increase in adipose tissue mass.

Studies have shown that high dietary sodium and insufficient potassium intake lead to an increased risk for CVD [27,28,29]. On the other hand, high potassium reduces the negative effect of high sodium on BP levels and, thus, the risk of CVD [30]. Despite the rigorous campaigns, discrepancies between actual and recommended sodium and potassium intake have repeatedly been reported [31,32]. Globally, the average dietary sodium intake is still double the recommended amount [25,33], and potassium intake is often suboptimal [34]. These data accurately reflect the situation in Croatia, as well; e.g., the average daily salt intake in Croatia in 2008 (salt mapping in Croatia—Croatian action on salt and health (CRASH)) was 11.6 g of salt/day (equivalent of 4640 mg of sodium per day), 13.3 g/day (5320 mg Na/day) for men and 10.2 g/day for women (4080 mg Na/day), which was more than double recommended daily amount [35]. Strategic Plan for Reduction of Salt Intake by the Croatian Institute of Public Health and the Ministry of Health, which was developed in order to reduce daily salt intake by 4% each year, according to data from 2019, resulted in a decrease in daily salt intake for 1.6 g of salt per day in a last 12 years (1.9 g of salt/day in men and 1.0 g of salt per day in women) [36]. Results of the current study have demonstrated that the median daily sodium intake in women in the third trimester of healthy pregnancy was between 2900 and 3000 mg of Na/day (24 h urine analysis: 2936 mg Na/day, FFQ: 2947 mg Na/day), which was below the Croatian average, but still considerably more than WHO recommendations for salt intake. Data on daily sodium intake in Croatian pregnant women could not be compared with data from other countries because results of large epidemiological studies on sodium intake were missing or did not extract data on sodium intake specifically for pregnant women.

Estimated daily potassium intake in healthy pregnant women shows some discrepancy depending on the method by which it was determined. Estimated potassium intake by 24 h urine analysis was much lower (average daily intake 2152 mg) when compared to the values obtained by FFQ (average daily intake 3399 mg), which once again underlines the complexity of determining the average daily nutrients intake by different methods (FFQ vs. 24 h urine collection). Thus, according to the 24 h urine analysis, daily potassium intake in pregnant women was much lower, while FFQ results indicate daily potassium intake close to recommended values by WHO (>3510 mg per day).

While in earlier large prospective studies, sodium and potassium intake were commonly assessed by FFQ, rather than the 24 h urine collection, dietary intake of sodium and potassium in recent studies is predominantly assessed by their 24 h urine excretion. Collection of 24 h urine over several days through the period of time was suggested as the accurate method for assessment of usual sodium (and potassium) intake, but studies reported that participants’ burden associated with repeated 24 h urine collection led to low compliance. Single-day measurement of 24 h urine collection can be useful for group intake assessment but has been shown as imprecise when assessing individual intake [37]. Bearing in mind all the weaknesses and strengths of the methods commonly used to estimate daily sodium and potassium intake, we used both FFQ and one-point 24 h urine collection analysis for the estimation of daily sodium and potassium intake in the present study. Public health surveillance studies have observed that sodium intake obtained by FFQ is often underestimated [38]. The present results show that, regarding daily sodium intake, average intake obtained from 24 h urine analysis and FFQ was approximately the same (24 h urine analysis: 3213 mg Na/day, FFQ: 3012 mg Na/day), suggesting that FFQ is validated. On the other hand, it is not clear why daily potassium intake was much lower when analyzing 24 h urine compared to FFQ data (24 h urine analysis: 2152 mg K/day, FFQ: 3399 mg Na/day). Thus, these results suggest that the variability in Na-to-K ratio obtained by 24 h urine and FFQ is entirely due to variability in K. On the other hand, estimated dietary intake assessed by FFQ yields low correlation coefficients for sodium and potassium with those of 24 h urine, which is consistently reported in previous studies [39].

In a recent year, the Na-to-K ratio was introduced as a surrogate indicator of sodium and potassium intake. Not only have studies shown that the Na-to-K ratio was related to BP, but it has been shown as a superior metric as compared to separate sodium and potassium for determining the relation to BP and CVD risks [40]. Na-to-K ratio as an index of sodium and potassium intake has been shown as independent of total energy intake [37,41], as well it is independent of 24 h urine collection accuracy or underreporting from FFQ. The present study reported results consistent with earlier reports [42,43], with healthy pregnant women having 24 h urine Na-to-K molar ratio values ranging from a minimum of 1.11 to a maximum of 5.24, which pointed out the poor fact that none of the 65 examined pregnant women had a Na-to-K ratio that met the recommendations of the WHO. Such a poor Na-to-K ratio resulted from both excessive sodium intake and insufficient potassium intake in pregnant women compared to WHO recommendations. The calculated Na-to-K molar ratio obtained by FFQ data was lower compared to one obtained from 24 h urine. Such a different FFQ Na-to-K molar ratio was attributed to higher daily potassium intake obtained by FFQ but with the results of 24 h urine analysis. These results indicate the necessity for the continuation of efforts aimed to acquire and improve basic knowledge and skills for sodium reduction and potassium increase in the everyday diet in the population in general, but with special attention given to the diet in pregnant women, children, and adolescent, and those with increased cardiovascular risk or already developed CVDs.

FFQ results obtained in the current study allowed the identification of food groups that are associated with a higher/lower Na-to-K ratio in healthy pregnant women, which could provide valuable data for strategies to change diet quality in this population. In the present study, the foods negatively associated with a lower dietary Na-to-K were ‘soups and sauces’, ‘cereals and cereal products’, as well as ‘fats and oils’, while the foods positively associated with a lower dietary Na-to-K ratio were ‘fruit’ and ‘non-alcoholic beverages’. Processed foods, such as cured and processed meat, breads, soups, and sauces, have been identified as key sources of sodium globally [44,45,46]. On the other hand, fruits, vegetables, dairy products, potatoes, and fresh meat are commonly reported as key dietary sources of potassium in adults [44,45,46]. The present study results are similar to findings from the Irish Adult National Survey and highlight the common determinant of both studies; soups and sauces, as well as fats and oils, contribute to a higher Na-to-K ratio, while fruit intake is associated with a lower ratio [47]. Poor individuals’ awareness of salt (sodium) intake [47] and ineffective individual efforts to reduce dietary salt [47] emphasizes the need for dietary recommendations on food groups and dietary patterns to provide adequate amounts of nutrients that are essential during pregnancy to promote health and prevent disease.

WHO guidelines on antenatal care for pregnant women include recommendations for several nutritional interventions during pregnancy, e.g., promotion of healthy eating and a physically active lifestyle to prevent excessive GWG; daily or intermittent use of iron and folate supplementation; calcium supplementation should be limited to populations with low-calcium intake; vitamin B6, zinc, multi-nutrient supplements, and vitamin D supplementation are not recommended as routine procedure; caffeine should be avoided [48]. The current study FFQ results indicate that the daily dietary intake of a substantial number of micronutrients (vitamin D, vitamin E, folate, iron, and zinc) is insufficient compared to the recommended dietary allowances (RDA) [7,49]. These results, consistent with other studies [50,51,52], support the use of iron and folic acid supplements during pregnancy, in addition to an iron- and a folate-rich diet, to prevent anemia and neural tube defects, respectively.

Even though some data indicates vitamin deficits during pregnancy [53], and around 20% to 30% of pregnant women worldwide suffer from some vitamin deficiency, there is no high-quality evidence demonstrating that all women require regular vitamin supplementation during pregnancy [7]. Moreover, despite WHO’s recommended calcium supplementation only in populations with low-calcium intake, and vitamin D use is not recommended as a routine, there is evidence that supplementation of vitamin D and calcium could be suggested during pregnancy to provide optimal maternal stores and to support fetal growth and bone development. In addition, vitamin D and calcium supplementation may prevent hypertensive disorders, especially in women with insufficient dietary intake or at high risk of deficiency [2]. N-3 polyunsaturated fatty acids (n-3 PUFA) intake is important during pregnancy because they can influence the development of the brain and retina in the fetus [54]. Studies have shown that n-3 PUFA supplementation during pregnancy prevents preterm birth [55]. Results on insufficient daily dietary intake of a substantial number of micronutrients (compared to RDA) during pregnancy underlie the need for clear, comprehensive, and widely applicable nutrition guidelines for pregnant women. Intake could be modified not only by supplements but also by introducing healthy dietary habits and consuming food naturally rich in micronutrients essential in pregnancy or through fortification programs of widely available food (e.g., salt iodization, vitamin D milk fortification, folate breads, and cereals fortification). Results of a number of studies investigating which maternal GWG is optimal for normal pregnancy outcomes yielded recommendations for weight gain in pregnancy based on pre-pregnancy BMI [6]. Both excessive or insufficient maternal GWG is associated with higher risks of adverse pregnancy outcome, including preterm birth, fetal macrosomia, large for gestational age (LGA), small for gestational age (SGA), gestational diabetes mellitus, preeclampsia, cesarean delivery, and obesity in childhood [49]. Within the scope of the current study, we examined the average pregnancy GWG, as well as its distribution according to the 2009 IOM classification. The results of a large systematic review and meta-analysis from 2018 on maternal and infant outcomes that involved more than one million women showed that GWG in Europe was above guidelines for 51%, within the guidelines for 31%, and below guidelines for 18% of examined pregnant women [15]. The current study showed that almost 47% of examined pregnant women with preconception BMI ≤25.1 kg/m^2^ had GWG classified as high, 38% had normal GWG, and 15% had GWG classified as low, which is consistent with the European average. Importantly, in participants with preconception BMI >25.1 kg/^2^, 77% of them had GWG classified as high, 17% had normal GWG, and 6% had GWG classified as low. Earlier studies demonstrated that GWG was positively associated with gains in fat mass (FM), but not fat-free mass (FFM) during pregnancy [56,57], which was confirmed by the results of the present study. Thus, these findings underline the fact that an increase in the proportion of adipose tissue during pregnancy is the most important determinant of GWG in pregnant women. Interestingly, GWG was not associated with the FFQ molar Na-to-K ratio, nor with total energy or any macronutrient intake (protein, carbohydrates, and fat). A possible explanation for this result is a poor self-awareness of food intake in general, which was supported by data that 42.2% of study participants classified as low energy reporters (LERs). Nevertheless, there remains a significant information on excessive weight gain during pregnancy in 47% (initial BMI ≤ 25.0 kg/m^2^) and 77% (initial BMI > 25.1 kg/m^2^) of examined women, respectively, mainly due to an increase in adipose tissue mass, which, along with guidelines for nutrition quality, support the necessity for increasing awareness and knowledge about optimal weight gain during pregnancy, contributing to the wellbeing of both the mother and the fetus.

## 5. Conclusions

The results of the present study indicate that the molar Na-to-K ratio assessed by both 24 h urine analysis and FFQ of healthy pregnant women in the third trimester of pregnancy does not meet recommendations set by WHO due to excessive sodium and insufficient potassium intake. In addition, FFQ results showed insufficient dietary intake of a substantial number of micronutrients (vitamin D, vitamin E, folate, niacin, riboflavin, calcium, iron, and zinc) compared to RDA. A total of 47% of initially underweight/normal-weight and 77% of initially overweight/obese examined pregnant women exhibited excessive GWG, which was attributed to the increase in adipose tissue mass, which was above the recommended values. Taken together, there is a need for more comprehensive, more intense, and more detailed nutrition counseling for all women during pregnancy. Moreover, implementation and awareness of the use of the Na-to-K ratio as an index of dietary quality, as well as instructions on how to achieve it, may lead to improvements in an individual’s ratio and may be a key factor for reaching the goals for dietary sodium and potassium intake set by WHO.

## Figures and Tables

**Table 1:** Participants’ characteristics, initial body mass index (BMI), and weight gain during pregnancy.

Characteristics				
Number	64			
Age (years)	29.4 ± 4.5			
Period of gestation (weeks)	37.9 ± 0.9			
Height (m)	1.68 ± 0.07			
Initial body mass index (kg/m^2^)	23.8 ± 4.3			
Body mass index classification	Underweight (BMI ≤ 18.5)	Normal (BMI 18.6–25.0)	Overweight (BMI 25.1–30.0)	Obese (BMI ≥ 30.1)
Number (%)	2 (3.1%)	45 (70.3%)	11 (17.2%)	6 (9.3%)
Gestational weight gain (kg) *	16.8 ± 5.4		15.1 ± 6.8	
Gestational weight gain classification †				
Number (%)	Low	7 (14.9%)	Low	1 (5.9%)
	Normal	18 (38.3%)	Normal	3 (17.6%)
	High	22 (46.8%)	High	13 (76.5%)

Data are presented as the mean ± standard deviation (SD). BMI- body mass index; GWG- gestational weight gain. * based on the participants’ weight measured at childbirth. † 2009 Institute of Medicine (IOM) Recommendations for gestational weight gain during pregnancy: BMI < 19.8 kg/m^2^ total weight gain between 12.5 and 18 kg; BMI = 19.8 to 26.0 kg/m^2^ total weight gain between 11.5 and 16 kg; BMI > 26.0 to 29.0 kg/m^2^ total weight gain between 7.0 and 11.5 kg. For BMI > 29.0 kg/m^2^, total weight gain of 7.0 kg [6].

**Table 2:** Healthy pregnant women body composition, body fluid status, and arterial blood pressure.

Characteristics	
Number of participants	65
Body mass index (kg/m^2^)	29.2 ± 4.6
RMR (kcal)	1678 ± 93
Fat-free mass (%)	68.6 ± 7.3
Fat mass (%)	31.5 ± 7.3
Total body water (%)	53.7 ± 6.2
Extracellular water (%)	45.1 ± 1.8
Intracellular water (%)	54.9 ± 1.8
ECW/ICW	0.82 ± 0.06
Plasma fluid (L)	4.07 ± 0.58
Interstitial fluid (L)	14.2 ± 2.0
Body density (kg/L)	1.03 ± 0.02
Systolic blood pressure (mmHg)	111 ± 9
Diastolic blood pressure (mmHg)	75 ± 6
Mean arterial pressure (mmHg)	87 ± 6.6

Data are presented as the mean ± standard deviation (SD). RMR—resting metabolic rate; ECW—extracellular water; ICW—intracellular water.

**Table 3:** Healthy pregnant women 24 h urine analysis.

Characteristics	
Number of participants	65
Creatinine coefficient (μmol/24 h/kg)	116 ± 34
Endogenous creatinine clearance	1.93 ± 0.54
Proteins (mg/dU)	202 ± 77
Albumin (mg/dU)	11 ± 11
Sodium (mmol/dU)	139.7 ± 46.8
Potassium (mmol/dU)	55.0 ± 20.7
Estimated daily sodium intake (mg)	3213 ± 1077
Estimated daily potassium intake (mg)	2152 ± 808
Estimated daily salt (NaCl) intake (g/day)	8.2 ± 2.7
Sodium-to-potassium ratio (molar ratio)	2.74 ± 0.91

Data are presented as the mean ± standard deviation (SD).

**Table 4:** Daily micronutrient intake and sodium-to-potassium ratio assessed by EPIC-Norfolk Food Frequency Questionnaire (FFQ) in healthy pregnant women.

Nutrient	RDA *	FFQ	FFQ vs. RDA
Vitamin A (μg/day)	770	880 ± 817	↔
Vitamin D (μg/day)	15	2.72 ± 1.29	↓
Vitamin E (mg/day)	15	12.1 ± 5.2	↓
Folate (μg/day)	600	257 ± 77	↓
Niacin (mg/day)	18	23.0 ± 6.6	↔
Riboflavin (mg/day)	1.10	1.85 ± 0.62	↔
Thiamin (mg/day)	1.4	1.55 ± 0.47	↔
Vitamin B_6_ (mg/day)	1.9	2.11 ± 0.63	↔
Vitamin B_12_ (μg/day)	2.6	6.49 ± 3.74	↑
Vitamin C (mg/day)	85	136 ± 76	↑
Sodium (mg/day)	<2000	3012 ±1015	↑
Potassium (mg/day)	>3510	3399 ± 938	↔
Calcium (mg/day)	1000	945 ± 308	↔
Iron (mg/day)	27	9.686 ± 3.17	↓
Phosphorus (mg/day)	700	1387 ± 392	↑
Selenium (μg/day)	60	102 ± 43	↑
Zinc (mg/day)	11	9.01 ± 2.72	↓
Sodium-to-potassium ratio (molar ratio)		1.51 ± 0.45	
Estimated daily salt (NaCl) intake (g/day)		7.53 ± 2.54	

Data are presented as the mean ± standard deviation (SD). * RDA—recommended dietary allowances [9,25].

**Table 5:** Daily energy and food group intakes in pregnant women assessed by EPIC-Norfolk food frequency questionnaire (FFQ).

	FFQ
EIrep (kcal/day)	1941 ± 638
Protein (% of total energy/day)	17.9%
Carbohydrates (% of total energy/day)	45.8%
Fats (% of total energy/day)	38.4%
EIrep/RMR	0.93 ± 0.32
LER, number (%)	27 (42.2%)
NonLER, number (%)	37 (57.8%)
Alcoholic beverages (g/day)	17 ± 42
Cereals and cereal products (g/day)	222 ± 104
Eggs and egg dishes (g/day)	24 ± 23
Fats and oils (g/day)	15 ± 8
Fish and fish products (g/day)	27 ± 23
Fruit (g/day)	330 ± 265
Meat and meat products (g/day)	157 ± 122
Milk and milk products (g/day)	391 ± 202
Non-alcoholic beverages (g/day)	455 ± 279
Nuts and seeds (g/day)	8.6 ± 8.7
Potatoes (g/day)	72 ± 42
Soups and sauces (g/day)	158 ± 83
Sugars (g/day)	49 ± 43
Vegetables (g/day)	200 ± 93

Data are presented as the mean ± standard deviation (SD). EIrep—reported energy intake from FFQ; RMR—resting metabolic rate; LER—low energy reporters.

## Data Availability

The data presented in this study are available on request from the corresponding author.
